# Effect of Negative Pulse on the Stability of Black Electrolytes for Magnesium Alloy Microarc Oxidation

**DOI:** 10.3390/ma17112654

**Published:** 2024-05-31

**Authors:** Bo Chen, Rui Tong, Hongtao Li, Wenqiang Wang, Xuanyu Chen, Hao Wang, Yifeng Yang, Shiquan Zhou

**Affiliations:** College of Materials Science and Engineering, Nanjing Tech University, Nanjing 211816, China; 202161203196@njtech.edu.cn (B.C.); 202261203248@njtech.edu.cn (R.T.); 202161203202@njtech.edu.cn (W.W.); 202161203207@njtech.edu.cn (X.C.); 202261203312@njtech.edu.cn (H.W.); 202261103119@njtech.edu.cn (Y.Y.); 202261203164@njtech.edu.cn (S.Z.)

**Keywords:** magnesium alloy, microarc oxidation, negative pulse, electrolyte stability

## Abstract

The correlation between negative pulse and the black electrolyte properties of magnesium alloy micro-arc oxidation and the treated area was investigated by introducing a negative pulse electric field. The physical phase composition, microstructure, elemental distribution, and content of the coating were analyzed using X-ray diffraction (XRD), scanning electron microscopy (SEM), and energy dispersive spectroscopy (EDS). The results showed that the introduction of negative pulses favored the generation of MgO and MgSiO_3_ contents in the coatings, and an increase in the MgO phase was found in the coatings formed in the failed electrolytes; the microporous size and microcracks of the coatings were gradually and significantly reduced; the average consumption of Cu ions was 0.0453 g/L·dm^2^, which is only 26% of that in the unipolar condition; the introduction of the negative pulses significantly improved the “anomalous consumption” of Cu ions. The introduction of negative pulse can significantly improve the “abnormal consumption” of copper ions, which is attributed to the change in the electric field by negative pulse, which makes the cathode-enriched Cu ions migrate to the anode and reduces the reduction and precipitation of Cu ions at the cathode.

## 1. Introduction

As representatives of lightweight materials, magnesium alloys are the lightest metallic materials for engineering applications, with a density that is only two thirds that of Al and a quarter that of Fe. Magnesium alloys have the advantages of low density, high modulus of elasticity, high specific strength and high specific stiffness, and recyclability [1,2,3], and are widely used in the fields of transportation, communication, medical treatment, and aerospace [4,5,6]. The use of magnesium alloys greatly reduces the weight of vehicles, enhances the aerodynamic performance of transportation vehicles, and contributes to energy conservation and emission reduction, impact resistance and vibration damping. Therefore, it is widely used in aerospace and transportation manufacturing industries. However, due to the high chemical activity of magnesium alloys, it has poor corrosion resistance, especially in marine environments [7]. AZ31 is a very commonly used aluminum-containing (or zinc-free) magnesium alloy in the world today, and its use offers the following engineering advantages: (a) low cost, (b) ease of handling, and (c) good strength (E = 44 ± 1 Gpa) and ductility (failure strain/elongation of 10.4 ± 3.9%) [8]. However, the low hardness (64 ± 4 Hv) [8] and low corrosion resistance (E_corr_: −1.35 mV, i_corr_: 8.54 × 10^−6^ A/cm^2^) of AZ31 magnesium alloy limit its wide application [9]. In recent years, with the development of technology in the field of aerospace, optical instruments, 3C electronic products, laser radar and other fields increasingly have requirements for the surface appearance color of magnesium alloy [10,11,12], and the requirements for magnesium alloy materials have gradually increased; corrosion-resistant, aging-resistant, non-fading magnesium alloy black coatings have become necessary for the development of magnesium alloy applications.

The surface treatment process of micro-arc oxidation with coloring can effectively improve the problems of poor binding and aging resistance of black pigments. The coating obtained by micro-arc oxidation technology can not only give magnesium alloy a black surface but can also greatly improve the corrosion resistance of magnesium alloy due to the principle of in situ companion coloring, which achieve the characteristics of strong bonding force, aging resistance and matting and light-absorbing properties.

However, regarding magnesium alloy micro-arc oxidation electrolytes, after long-term use, the electrolytes will become turbid from clear or even white flocculent precipitation. In this kind of electrolyte, to continue the micro-arc oxidation treatment, it is difficult for the specimen surface to form a complete micro-arc oxidation coating; at this time, the electrolyte state is considered a failure.

For the study of white electrolyte failure of magnesium alloys, it was found that the changes in pH and conductivity of the electrolytes were important for electrolyte failure. With the increase in the treated area, the roughness of the micro-arc oxidized coating increased, the film thickness decreased, and both pH and conductivity showed a gradual decrease [13]. And the stability of magnesium alloy electrolytes is mainly related to the OH^−^ concentration [14]; the lower the OH^−^ content in the electrolytes, the more stable the magnesium alloy is; at the same time, the electrolytes are also related to the air contact, and the addition of sodium hexametaphosphate to failing electrolytes by Tai et al. [14] prolonged the service life of the electrolytes. The ion concentration in the electrolytes has a greater effect on the electrolyte stability, and the degree of influence is in order from largest to smallest: OH^−^ > F^−^ > SiO_3_^2−^, i.e., the concentration of OH- has the greatest influence on the failure of the electrolytes [15]. In the study by Hou [16], it was pointed out that the precipitates in the electrolytes gradually increased with the increase in the treated area, the thickness of the coating gradually decreased, and the corrosion resistance decreased. It is difficult to form a film on the surface of the magnesium alloy substrate after the failure of the electrolytes. The anion concentrations in the solution all decreased with the increase in the treated area, in which the concentration of SiO_3_^2−^ changed the most. The precipitates in the electrolytes were analyzed and detected; the results showed that the precipitates in the electrolytes were generated by the reaction of Mg^2+^ escaping from the oxidation process with OH^−^, SiO_3_^2−^ and F- in the solution, and the generation of these precipitates impeded the migration of ions and suppressed the generation of MgO, which resulted in the failure of the surface of magnesium alloy to form a film.

However, there are fewer studies on the stability of magnesium alloy black electrolytes, and the form of failure of magnesium alloy black electrolytes is also different from that observed in white electrolytes. When using aluminum alloy, black electrolytes also tend to fail easily; the failure manifests as difficulty in forming a black coating on the surface of the aluminum alloy. Research shows that NH_4_VO_3_ as the coloring salt, whether under acidic or alkaline conditions, will decompose in different forms during the micro-arc oxidation process, which will break the stability of the electrolyte system, and at the same time, reduce the pH value, increase the K value, and make the electrolytes fail. Impurity ions, such as Fe^3+^, Cu^2+^, etc., are very difficult to migrate to the surface of the anode in a high-voltage field, and the contribution of the impurity ions to electrolyte failure is thus very small and can be ignored. Therefore, the failure of the micro-arc oxidized black coating electrolytes can be attributed to the decomposition of NH_4_VO_3_, which breaks the equilibrium of the electrolyte body.

However, in the process of magnesium alloy micro-arc oxidation, when Cu salt is used as a colorant, it is difficult to ignore the change rule of Cu^2+^ in the process of micro-arc oxidation. However, no study has explored the failure of black electrolytes for magnesium alloy. In this paper, we examine the reason for Cu^2+^ “consumption abnormality” from the regulation of the inter-polar electric field, we and explore the reason for the improvement of the stability of magnesium-based black electrolytes for micro-arc oxidation.

## 2. Details of Experiment

### 2.1. Experiment Preparation

In this paper, the common AZ31 die-cast magnesium alloy is used as a base material. The specimens used in the experiments were 40 mm × 40 mm × 4 mm rectangular AZ31 magnesium alloy (3.12% Al, 1.04% Zn, 0.001% Fe, 0.001% Cu, 0.44% Mn, 0.06% Si and balance Mg). Prior to the experiment, the surface of the specimens was sanded to an average roughness of 0.1 µm using SiC sandpaper, and then the sanded specimens were placed in anhydrous ethanol and ultrasonically cleaned using an ultrasonic cleaner in order to remove the surface oils. The specimens were rinsed with deionized water and blown dry before use.

**Figure 1:**
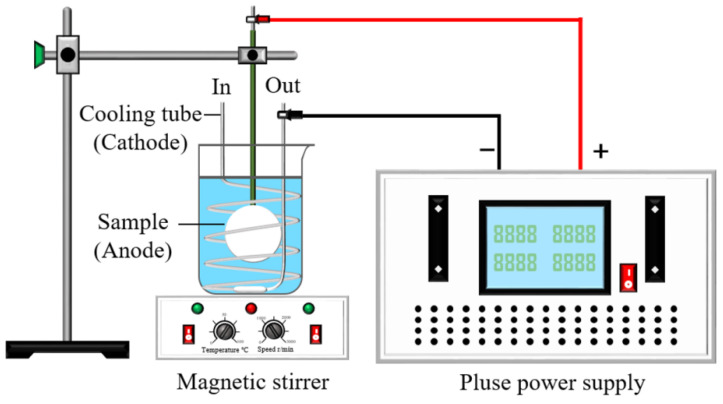
Process schematic of micro-arc oxidation.

The following electrolyte formulations and electrical parameters were determined based on the results of extensive pre-experimentation. The electrolytes used in this paper consisted of Na_2_SiO_3_, KF, NaOH, Cu_2_P_2_O_7_ and KNaC_4_H_4_O_6_ with a content ratio of 8:10:8:5:5. The electrical parameters of unipolarity were as follows: duty cycle of 10%, frequency of 800 Hz, current density of 2 A/dm^2^, and oxidation time of 10 min; the electrical parameters of bipolarity were as follows: duty cycle of 10%, frequency of 800 Hz, current density of 2 A/dm^2^/−1.25 A/dm^2^, oxidation time of 10 min, and electrolyte temperature control of approximately 30 °C. The coatings prepared before and after the failure of the electrolytes under unipolar and bipolar conditions were examined with a coating L-value greater than 30 as the failure criterion and were recorded as D1, D2, D3, and D4, respectively. The process schematic of micro-arc oxidation is shown in Figure 1.

### 2.2. Characterization of Coatings

**Figure 2:**
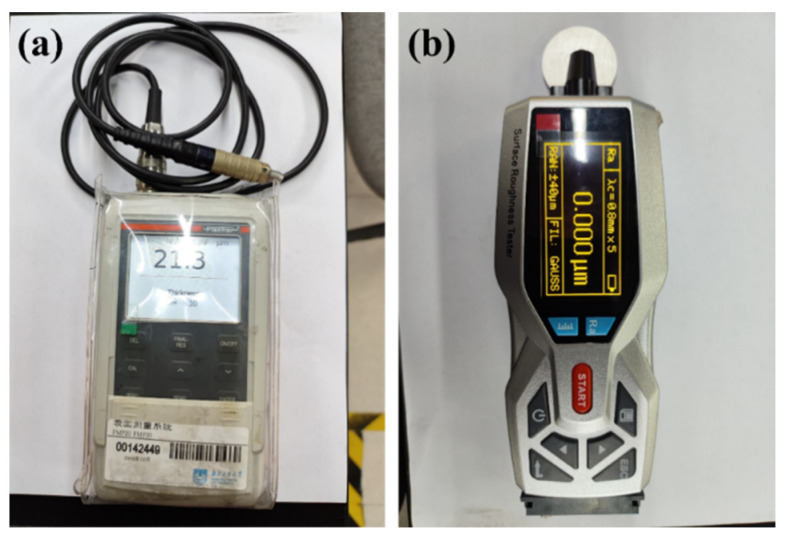
The applied (**a**) eddy current coating thickness gauge and (**b**) surface roughness tester.

The resulting micro-arc oxidized coatings were measured using an eddy current thickness gauge, model FMP20 (Figure 2a). Five points were measured on each of the two sides of the specimen and the measured values were averaged. The measuring principle of the eddy current thickness gauge is to use the size of the eddy current generated between the metal probe and the base metal to assess the thickness of the coating; higher eddy current feedback results indicate that the closer the distance between the probe and the substrate, the smaller the thickness of the non-conductive coating shown, and vice versa. Roughness was detected using an Ra200 roughness tester (Figure 2b). The measurement process requires placing the probe to detect the position and then adjusting the roughness meter to the appropriate level to ensure the accuracy of the test results. Three points were tested on each side of the sample, and the average value was taken as the final test data.

The physical phase composition of the micro-arc oxidation coatings was analyzed using grazing incidence X-ray diffraction (XRD, D/Max-2400, Akishima, Japan). A Cu target (Kα1 = 0.15406 nm) was selected as the anode target, the scanning rate was 10°/min, the grazing incidence angle was set to 4°, the scanning range was set to 10~90°, and the step size was set to 0.02°. The test specimen was selected from the central region whenever possible.

Scanning electron microscopy (SEM, JEOL, JSM-7900F, Akishima, Japan) was used to examine the surface morphology of the micro-arc oxidation treated samples, and the elemental composition of the coatings was analyzed and characterized using energy chromatography (EDS, JEOL, JSM-IT500A, Akishima, Japan). To avoid the occurrence of the edge effect, the surface samples were selected from the middle part of the specimen and cut into small pieces of 10 mm × 10 mm, and the edge burrs were polished and then placed in anhydrous ethanol for ultrasonic cleaning for 10 min. The cross-section samples were also selected from the center area, polished, and then placed in a mounting tank, and they were then cured and mounted with epoxy resin. The samples were then polished with SiC abrasive paper from low to high, and the polished samples were rinsed with a large amount of deionized water. A metallurgical microscope was used to observe the polished section before testing, and a scanning electron microscope (SEM) was used to characterize the section after it was observed to be clean and free of obvious scratches. The surface morphology of the samples was observed in secondary electron mode, while the cross-section morphology was observed in backscattering mode. Since the micro-arc oxidized coating is an insulating layer, it is necessary to pre-spray the surface cross section for inspection.

An LS171 colorimeter was used to detect the blackness of the micro-arc oxidized coatings. The instrument was placed vertically on the surface of the sample to be tested, the test button was pressed, and the instrument measured the results. In order to minimize the experimental error, the two surfaces of the resulting samples were measured three times to take the average value. The evaluation of the coating color was calculated based on the L*a*b* standard chromaticity developed by the International Commission on Illumination, where L* represents black and white (0~100), a* represents green-red (−128~128), and b* represents blue-yellow (−128~128). The larger the L*, the whiter the coating color, and smaller the L*, the blacker the coating color; the more positive the a*, the redder the coating color, and the more negative the a*, the greener the coating color; the more positive the b*, the more yellow the coating color, and the more negative the b*, the bluer the coating color; Lab values of 0 indicate pure black.

**Figure 3:**
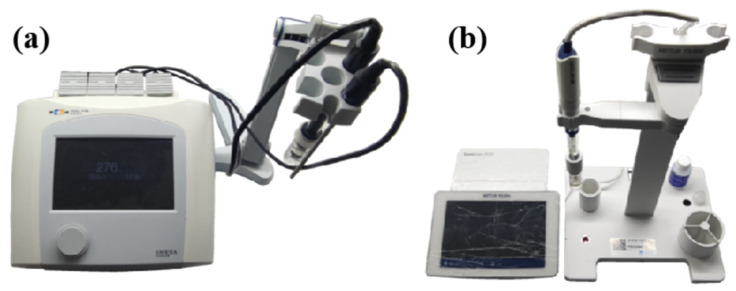
(**a**) Conductivity meter; (**b**) pH meter.

After the configured black electrolytes of magnesium alloy were fully stirred and homogenized, a conductivity meter was used to measure the conductivity of the solution (Figure 3a). In order to ensure the accuracy of the test results, the test probe was first immersed in potassium chloride solution and then immersed in the electrolytes to be measured after the residual potassium chloride was sucked up with a dust-free cloth, and then the data were recorded after the detection value was stabilized, and the error was reduced by multiple measurements. When measuring the pH value of the electrolytes, it was first calibrated with the calibration solution, and the detection method is similar to the conductivity detection (Figure 3b). 

In this experiment, an ICP-OES/MS (inductively coupled plasma emission spectrometer) was used to test the content of elemental Cu in the electrolytes; the concentration of the measured element needed to be diluted to less than 10 ppm before measurement, and the test was conducted for 2–6 min for each sample.

## 3. Results and Discussion

### 3.1. Growth Characteristics of Magnesium Alloy Microarc Oxidation Coatings

#### 3.1.1. Effects of Different Power Supply Modes on the Growth Characteristics of Magnesium Alloy Microarc Oxidized Coatings

**Figure 4:**
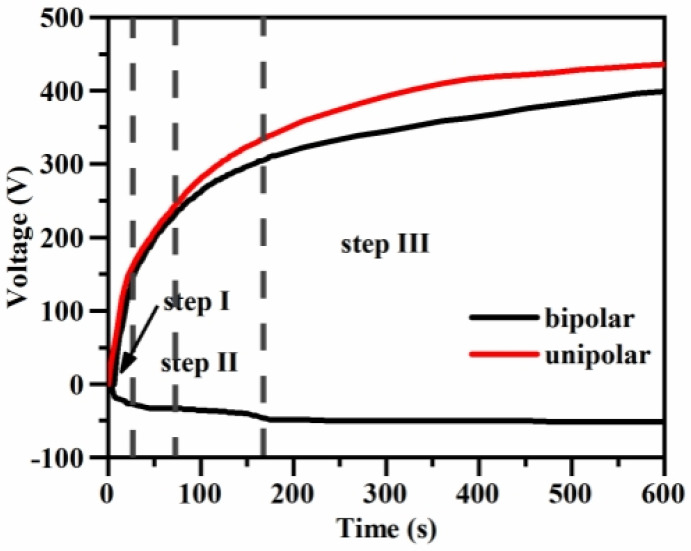
Time–voltage curves of the micro-arc oxidation process under different power modes.

Figure 4 shows the time–voltage curves of the microarc oxidation process under different power modes. It can be seen that compared with unipolarity, after the introduction of negative pulse, the forward voltage starts to decrease after 72 s of microarc oxidation, with a maximum decrease of approximately 60 V, and does not exceed 400 V in the middle and late stages of the coloring film formation after 320 V (5 min). The introduction of negative pulse can effectively reduce the forward voltage and inhibit the large arc discharge in the forward direction. This also resulted in a consistently lower forward voltage in the bipolar mode than in the unipolar mode.

**Figure 5:**
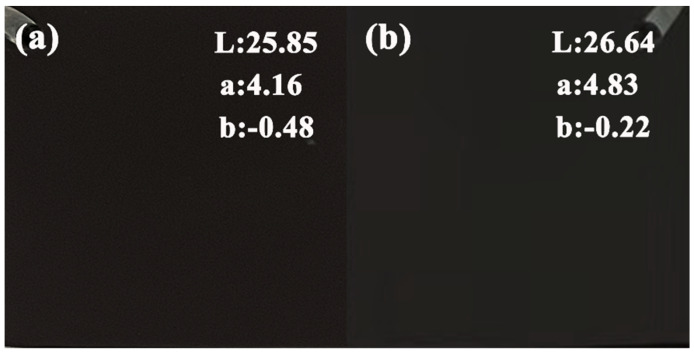
Macroscopic surfaces of micro-arc oxidized coatings formed in different power modes.

The macroscopic surfaces and L-values of the micro-arc oxidized coatings formed in unipolar and bipolar modes are shown in Figure 5. It can be seen that the introduction of negative pulses reduces the black value of the coating, making the L value increase, which is similar to the findings of Bai et al. [17], where the introduction of negative pulses reduces the intensity of the positive sparks, which reduces the solid solubility of Cu^2+^, leading to a lighter color of the coating, thus increasing the L value. Although the introduction of negative pulses increases the L of the coating, a black coating with good appearance can still be obtained, which can satisfy the requirements of industrial production for black coatings. Therefore, the introduction of negative pulses can be used to improve the stability of the electrolytes.

#### 3.1.2. Effect of Treatment Area on the Growth Characteristics of Magnesium Alloy Microarc Oxidized Coatings in Different Power Modes

**Figure 6:**
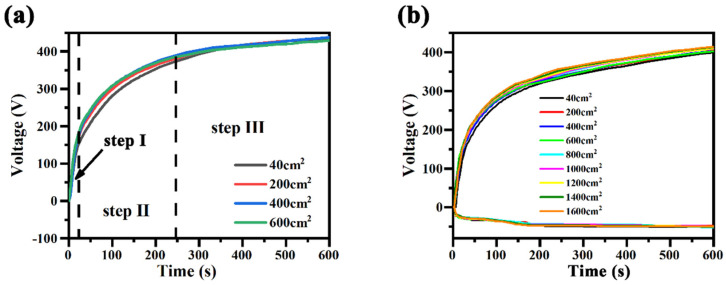
Influence of treatment area in different power modes on the curves of voltage response time during the micro-arc oxidation of magnesium alloys: (**a**) unipolar, (**b**) bipolar.

During micro-arc oxidation treatment, different power supply modes were used, and the variation curves of the voltage response time during the experiment were recorded, as shown in Figure 6. From Figure 6a, it can be seen that the effect of the treatment area in the unipolar mode on the time–voltage profile during the microarc oxidation of magnesium alloy is not significant and remains almost constant. In the early stage of micro-arc oxidation, the oxidation voltage rises rapidly, and under the action of an electric field, various anions in the electrolytes migrate rapidly to the anode surface and are deposited, while another part of the precipitation can also reach the anode surface under the action of stirring and participate in the formation of passivation film. The migration of ions is affected by the conductivity of the electrolytes, and the ease of migration of ions determines the rate of formation of passivation film, and it also determines the oxidation voltage. Further thickening of the passivation film improves the resistance of the coating, so that the working voltage continues to increase; the discharge intensity also increases, and the micro-arc oxidation coating is formed in the “breakdown-film” repeated process. Repeated breakdown discharge facilitates the formation of micro-arc oxidation coating.

Since only a single electric field exists under unipolar pulse conditions, the migration of ions in the microarc oxidation process is mainly affected by the electric field, but the anion species in the magnesium alloy electrolytes are complex, and the migration rates of the anions driven by the electric field are also different, so it is difficult to regulate the concentration gradient of the anions at a constant electric field. While the magnesium alloy for the microarc oxidation coating is mainly composed of magnesium-based metal oxides and metal ion oxides contained in the solution, the varying anion migration rates lead to a competitive relationship during the formation of magnesium-based microarc oxidation black coating. This competitive relationship occurs in the process of anion formation, particularly in the interface electrochemical/plasma discharge interaction, resulting in the formation of magnesium-based microarc oxidation black coatings with different compositions and structures. Meanwhile, the presence of Cu precipitates was found in the electrolytes after standing, and a large amount of purple-red copper was observed on the surface of the stainless-steel cathode coil. These various reasons led to the “abnormal depletion” of Cu in the electrolytes, which resulted in a reduction in the life of the electrolytes to only 10% of the white electrolytes.

And the effect of the treatment area on the time–voltage profile of the magnesium alloy microarc oxidation process in the bipolar mode exhibits the same rule of change. However, due to the introduction of negative pulse, it has a certain inhibitory effect on the growth of positive voltage, which is the reason why the termination voltage in the bipolar mode is lower than that in the unipolar mode. Unlike the white electrolytes, which cannot form a film state after failure, with the failure of the black electrolytes of magnesium alloy, they can still form a complete coating.

**Figure 7:**
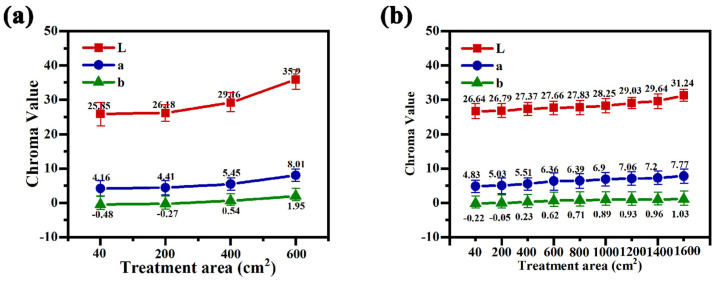
Evolution of the Lab value of the black coating of magnesium alloys in different power modes: (**a**) unipolar, (**b**) bipolar.

Figure 7 shows the evolution of Lab values of the black coating of magnesium alloy in different power modes. From the figure, it can be seen that the Lab values of the coatings all show a slow increasing trend with the increase in the treated area, which more intuitively shows the evolution of the coating color with the increase in the treated area. Figure 7a depicts the trend in Lab value of the black coating of magnesium alloy in the unipolar mode, and it can be seen that, with the increase in the treated area, the L value of the coating increases from the initial 25.85 to the final 35.9, and at this time, the treated area is only 6 dm^2^; additionally, it can be seen that, according to the failure criterion of the magnesium alloy black coating of micro-arc oxidation defined earlier, the electrolytes are in the failure state at this time. In the bipolar mode, with the increase in the treatment area, the L value of the micro-arc oxidized coating increased from the initial 26.64 to 31.24, and at this time, the treatment area was 16 dm^2^, and the treatment area increased by 166.7%, which greatly improved the stability of the electrolytes. The a value and the change rule of the b value indicate that, with the increase in the treatment area, the color of the coating gradually departs from the black color and moves towards red and yellow color. Compared with the results of Hou [16], the maximum treatment area of 2L of the white electrolytes before failure is 28 dm^2^, and the maximum treatment area of the black electrolytes in unipolar mode is only 21% of the life of the white electrolytes, while the maximum treatment area of the black electrolytes in bipolar mode can reach 57% of the life of the white electrolytes.

**Figure 8:**
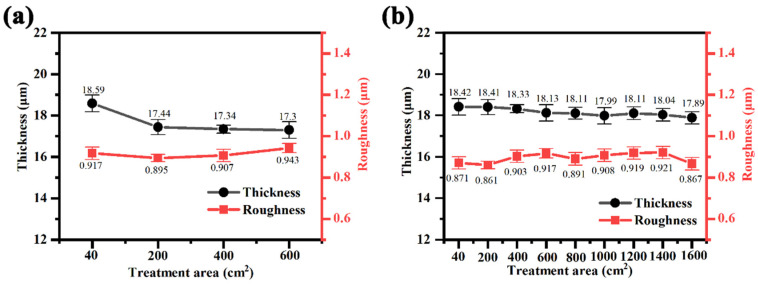
Effect of treatment area on film thickness and roughness of magnesium alloy micro-arc oxidized coatings in different power modes: (**a**) unipolar, (**b**) bipolar.

Figure 8 shows the thickness and roughness of the micro-arc oxidized coatings under the treated area in different power modes. The results show that the film thickness of the magnesium alloy micro-arc oxidized coating shows a gradual decrease with the increase in the treated area in both unipolar and bipolar modes. And the change in roughness was influenced by the coating thickness, on the one hand, and the measurement error, on the other hand. When the film thickness is not much different, the change in roughness is in the range of normal fluctuation. In the unipolar mode, when the treated area reaches 6 dm^2^, the thickness of the coating decreases from the initial 18.59 μm to 17.3 μm, which is 1.29 μm or 6.9%; in the bipolar mode, the film thickness of the micro-arc oxidized coating formed by treating 16 dm^2^ is 17.89 μm, which is only 0.53 μm lower than that of the initial film thickness of 18.42 μm. In summary, the results show that the black electrolytes of magnesium alloy in bipolar mode exhibit better stability, both in terms of the treated area before failure and the change in the thickness of the micro-arc oxidation coating after failure.

### 3.2. Influence of Phase Composition and Microstructure of Microarc Oxidized Coatings before and after Electrolyte Failure under Different Power Modes

**Figure 9:**
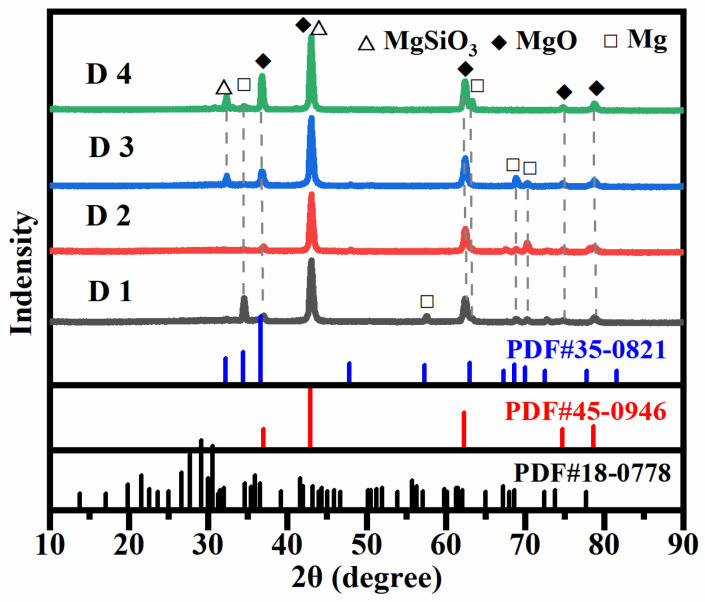
Physical phase composition of coatings obtained after microarc oxidation treatment before and after electrolyte failure in different power modes.

Figure 9 demonstrates the physical phase composition of the coatings obtained through micro-arc oxidation treatment before and after electrolyte failure in different power modes. It can be seen from the figure that the micro-arc oxidized coating is mainly composed of Mg, MgSiO_3_ and MgO, and no compounds of Cu were detected in the coating, for the reasons explained in the previous section. It can be seen in the figure that the peak intensities of MgO and MgSiO_3_ in the coating in the bipolar mode are significantly higher than those in the unipolar mode, which suggests that the introduction of negative current can promote the formation of MgO and MgSiO_3_. In addition, the XRD results show that the coatings in the electrolytes after failure exhibit an increase in the peak intensity of MgO, which indicates that the decrease in the content of Cu elements has a facilitating effect on the generation of MgO.

**Figure 10:**
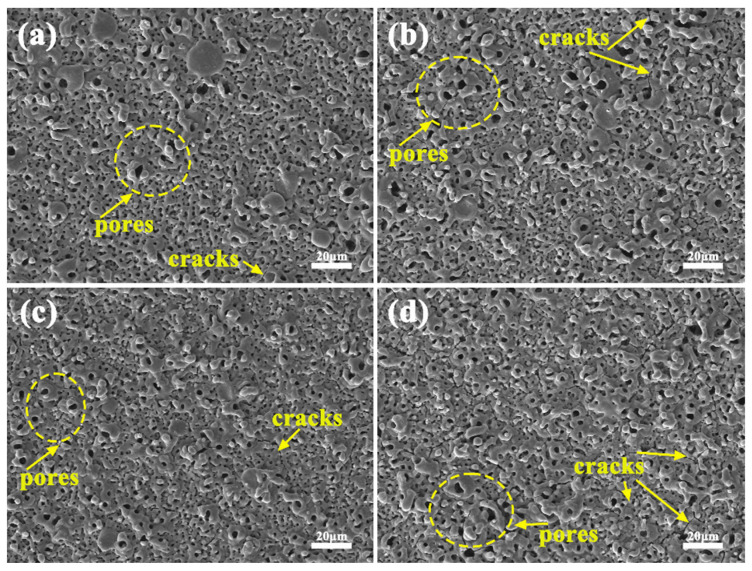
Surface morphology of coatings obtained after micro-arc oxidation treatment before and after electrolyte failure in different power modes: (**a**) D1, (**b**) D2, (**c**) D3, (**d**) D4.

Figure 10 demonstrates the surface morphology of the coatings obtained by micro-arc oxidation treatment before and after electrolyte failure in different power modes. Microcracks and discharge channels can be observed on the surface of all coatings, and micropores in oxide coatings usually arise from random discharge channels and gas trapping [18], which are irregular holes of different sizes generated by discharge breakdown and bubble film rupture [19]. The micropores are surrounded by the accumulation of molten oxides “ejected” from the discharge channel, showing a “crater” organization, with a dense “skeleton” connecting the pores to each other. Microcracks are usually attributed to the difference in thermal conductivity between the rapidly cooling molten oxide and the magnesium alloy matrix [20]. Such surfaces exhibit a mesh or scaffold structure, and sintered structures are also present in the coating [21,22]. The surface morphology of the micro-arc oxidized coating before and after electrolyte failure in the unipolar mode is shown in Figure 10a,b. It can be seen that the surface of the micro-arc oxidized coating after failure is still porous in nature and there are a large number of micro-cracks, but the size of the micro-holes and the number of cracks are significantly increased compared with that before failure, which may be related to the strong discharge caused by the reduced conductivity of the electrolytes after failure. The micro-arc oxidized oxide coatings formed in the bipolar mode have significantly smaller micro-pore sizes and fewer micro-cracks compared to the unipolar ones. This is, on the one hand, related to the lowering of the barrier (dielectric breakdown) potential by the cathodic pulse. On the other hand, the presence of negative pulses leads to a change in the electric field, anodic hydrogen precipitation facilitates the transfer of electrons to the oxide layer, and H^+^ in the discharge channel raises the plasma temperature, facilitating the conversion of magnesium hydroxide complexes to hydroxides and oxides at these sites. The micro-arc oxidation process is effective in repairing defects and sealing micropores [23]. The micro-arc oxidized coatings formed in the electrolytes after failure in the bipolar mode also exhibited similar surface structures compared to the coatings formed after failure of the unipolar electrolytes. In summary, it is known that the introduction of negative current reduces the porosity of the coatings to a certain extent, and the surface porosity of the micro-arc oxidized coatings formed in the post-failure electrolytes increases and cracks increase.

Table 1 demonstrates the surface EDS elemental results of the micro-arc oxidized coatings formed before and after electrolyte failure in different power modes. From the figure, it can be seen that the micro-arc oxidized coatings are mainly composed of the elements Mg, O, F, Si and Cu. The Mg element comes from the base metal, and the rest of the elements come from the electrolytes. It is clearly seen that the content of the Cu element on the surface of the micro-arc oxidized coatings formed in the failed electrolytes is significantly reduced, and the Cu element is almost undetectable in the coatings formed in the unipolar mode, whereas, in the bipolar mode, there is still 0.44% remaining. This also indicates that the bipolar mode reduces the consumption of elemental Cu in the solution and prolongs the stability of the electrolytes.

**Figure 11:**
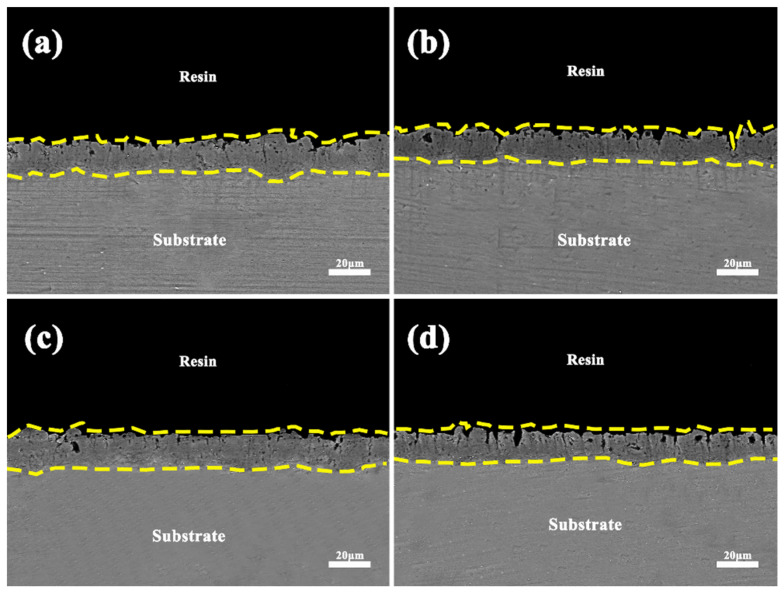
Cross-sectional morphology of coatings obtained after micro-arc oxidation treatment before and after electrolyte failure in different power modes: (**a**) D1, (**b**) D2, (**c**) D3, (**d**) D4.

Figure 11 shows the cross-sectional morphology of the coatings obtained after microarc oxidation treatment before and after electrolyte failure in different power modes. From the figure, it can be seen that all the coatings are composed of an outer porous and loose layer and an inner dense layer. The thickness variation in the coatings can be explained by the discharge inhomogeneity, which is due to the localized continuous thickening of the coatings caused by the discharge breakdown. In addition, different types of discharges and thermal stresses caused microcracks and pores. According to the discharge model proposed by Cheng et al. [24], five types of discharges may occur during the microarc oxidation process: type B discharges occur between the substrate and the coating, type A discharges occur at the oxide/electrolyte interface or bubbles attached to the coating surface, type C discharges are located at the oxide/electrolyte interface within the pores and cracks, type D discharges occur as bubbles in the coating pores, and type E discharges occur at the deep channels at the interface between the inner and outer oxide layers. As can be seen in the figure, the most common types of discharges are A, C, D and a small number of E-type discharges. No discharge channels through to the substrate are found in the figure, which indicates that B-type discharges do not occur.

Compared to unipolarity, in the unfailed electrolytes, the thickness of the coating was more uniform and less porous in the bipolar condition, which was attributed to the introduction of negative pulses that suppressed the strong discharge in the positive direction and reduced the porosity of the coating. In the failed electrolytes, the coating exhibits irregular jaggedness of the oxides/electrolytes, which is due to the high porosity caused by the strong discharge resulting from the reduced conductivity and stability of the electrolytes, which is also the reason for the high surface porosity.

**Figure 12:**
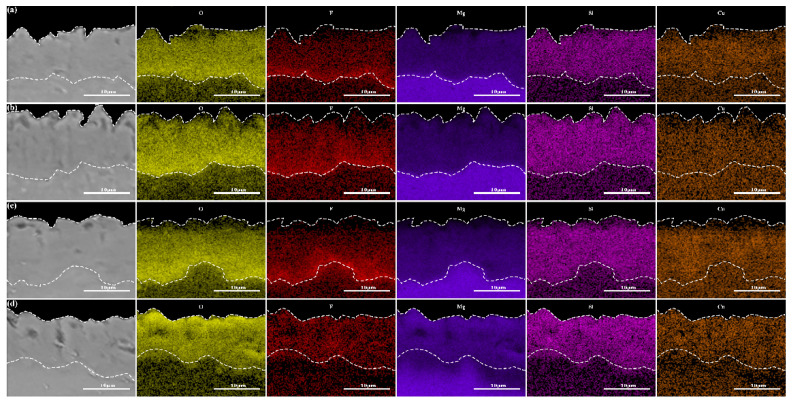
Cross-sectional EDS elemental distributions of micro-arc oxidized coatings: (**a**) D1, (**b**) D2, (**c**) D3, (**d**) D4.

Figure 12 demonstrates the cross-sectional EDS elemental distribution of the micro-arc oxidized coatings formed before and after electrolyte failure in different power modes. From the figure, it can be seen that the micro-arc oxidized coatings are mainly composed of Mg, Si, F, O and Cu elements. The Mg elements are derived from the substrate magnesium alloy, and the rest of the elements are from the electrolytes. The F elements are enriched at the substrate/oxide, which is due to the fact that the F ions migrate more readily to the inside of the coatings and react with the Mg^2+^ to form the MgF_2_, whereas the rest of the elements are distributed homogeneously in the coatings. The Cu element in the coating formed in the failed electrolytes is significantly lower, indicating that the amount of Cu element in the solution involved in film formation is significantly reduced, which also indicates that the electrolytes are in a failed state at this time.

### 3.3. Effect of Treatment Area on Electrolyte Base Properties in Different Power Modes

**Figure 13:**
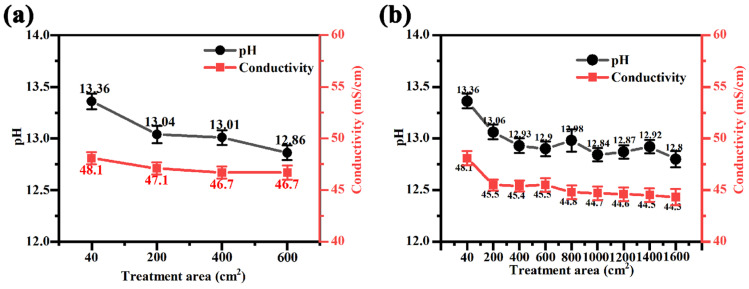
Changes in pH and conductivity of silicate electrolytes with increasing treatment area in power mode: (**a**) unipolar, (**b**) bipolar.

Figure 13 demonstrates the changes in pH and conductivity of silicate electrolytes with an increasing treatment area in different power modes. From the figure, it can be seen that the pH and conductivity of silicate electrolytes show a gradual decrease with the increase in treatment area in both unipolar and bipolar mode. The solution conductivity reflects the conductive ability of the solution and is also an important indicator of the total amount of free ions in the reaction solution, while the solution pH is related to the free OH^−^. The decrease in pH and conductivity indicates that during the process of magnesium alloy micro-arc oxidation, some of the ions in the solution are involved in the generation of the micro-arc oxidation coatings; these ions interact to generate a colloid or a precipitate, and the depletion of these ions causes decrease in the total ionic concentration in the electrolytes, resulting in a decrease in the conductivity of the electrolytes.

The elemental Cu content in the electrolyte solution before and after failure was determined using an ICP-OES Thermo Fisher iCAP PRO inductively coupled plasma emission spectrometer. Table 2 lists the elemental Cu content in the solution before and after failure of the electrolytes. According to the calculation, a total of 2109 mg/L of Cu was added to the solution, and the test results showed that there was a total of 1495 mg/L of Cu in the initial electrolytes, indicating that the introduced Cu was not completely complexed to form Cu complex ions, and some of the Cu reacted with the electrolytes to form Cu(OH)_2_ precipitates. It can be clearly seen from Table 2 that the elemental Cu in the electrolytes after failure in the unipolar mode was only 448 mg/L, and after 6 dm^2^ of treatment, the elemental Cu content was reduced by 70.0%, with an average consumption of 174.5 mg/L of elemental Cu for every 1 dm^2^ of the sample treated; in the bipolar mode, the content of elemental Cu in the electrolytes remained at 770 mg/L, and Cu(OH)_2_ precipitation was formed by the chemical reaction between some Cu and the electrolytes. Specifically, the content of elemental Cu was only reduced by 48.5%, and an average of 45.3 mg/L of elemental Cu was consumed per 1 dm^2^ of sample treated. The Cu element consumed in the unipolar mode was 3.85 times that in the bipolar mode. Meanwhile, it can be illustrated that the stability of the electrolytes was improved by 2.85 times in the bipolar mode, greatly prolonging the service life of the electrolytes.

### 3.4. Failure Analysis

**Figure 14:**
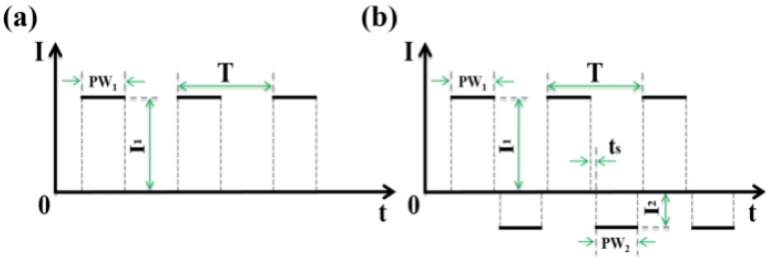
Output waveform of micro-arc oxidation power supply: (**a**) unipolar, (**b**) bipolar.

The output waveforms of the power supply in unipolar and bipolar modes are shown in Figure 14. In the bipolar mode, the positive and negative pulses alternate with the negative pulse, and there is a toff interval during the alternation of positive and negative pulses, in which the power supply is not discharged to provide a buffer for the next stage of discharge, where T is a period, PW1 is the pulse width of the positive pulse, and PW2 is the pulse width of the negative pulse. The electric field changes when the positive and negative pulses alternate.

**Figure 15:**
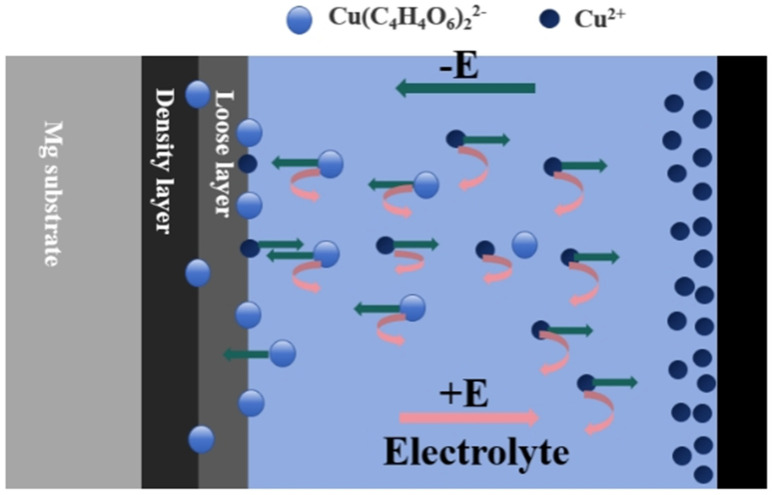
Schematic diagram of the movement of ions during micro-arc oxidation in bipolar mode.

Figure 15 explains the reason for the differentiation of electrolyte life in different power modes by the movement of ions in the electrolytes. In the microarc oxidation process, the positive and negative electric fields shift with the alternation of positive and negative currents. When the positive pulse is in operation, the migration rate of anions migrating to the anode surface to participate in film formation varies due to the more complex composition of the electrolytes and the wide variety of anions. Compared to other anions, the migration of the large molecule Cu(C_4_H_4_O_6_)_2_^2−^ driven by the electric field is relatively difficult; therefore, only a small amount of elemental Cu is detected during the formation process in the coating (Table 1). However, due to the presence of Cu oxides, it is hypothesized that Cu(C_4_H_4_O_6_)_2_^2−^ can be decomposed to produce Cu ions during the microarc oxidation process, and some of the Cu ions participate in the plasma reaction to form magnesium-based microarc oxidized black coatings, while other Cu ions migrate to the cathode coil under the driving force of the electric field, and at this time, the migration of Cu ions is much easier compared to Cu(C_4_H_4_O_6_)_2_^2−^, and they can easily participate in the coating formation process (Table 1). They can thus much more readily participate in the electrochemical reaction at the cathode, precipitating to form Cu monomers. During the positive pulse toff, the migration of ions in the electrolytes is partly affected by the ion concentration gradient, while the other part is rearranged by agitation or convection. When the negative pulse works, the incompletely consumed Cu ions again diffuse rapidly to the magnesium alloy substrate under the action of the electric field, while the anodic hydrogen precipitation increases the temperature of the discharge channel, which promotes the participation of Cu ions in the film-forming process of the magnesium-based micro-arc oxidized black coating. Although Cu(C_4_H_4_O_6_)_2_^2−^ is transformed by the electric field and migrates to the cathode coil, the migration rate is slow; moreover, at this time, a large number of anions are gathered near the cathode coil, and Cu(C_4_H_4_O_6_)_2_^2−^ is almost not involved in the consumption of reaction at the cathode. And during the toff of the negative pulse, the ions in the solution are similarly affected by stirring convection and the concentration gradient. During the continuous uninterrupted interfacial electrochemical/plasma discharge interaction, the magnesium-based microarc oxidized black coating was gradually formed. Only a few flocculent or colloidal precipitates appeared in the electrolytes after standing, and the stability of the electrolytes was significantly improved.

## 4. Conclusions

This paper focuses on the effect of the power mode on the stability of magnesium alloy black electrolytes. The physical phase composition and microstructure of the coating formed in the electrolytes before and after failure were analyzed and discussed, and the fundamental characteristics of the electrolyte evolution were examined; additionally, the rationale for the improvement of the anomalous consumption of the Cu element by the introduction of negative pulses was analyzed, and the failure of the black electrolytes in terms of the coating L > 30 was found to be as follows:

(1) After the introduction of negative pulse, the content of MgO and MgSiO_3_ in magnesium-based black microarc oxidized coatings increased, and the enhancement of MgO phase was found in all the coatings formed in the failed electrolytes; the microporous size and microcracks of the coatings were gradually and significantly reduced.

(2) After the introduction of negative pulse, in the process of film formation of a magnesium-based micro-arc oxidized black coating, the life of the electrolytes can be improved by threefold, which is approximately 8 dm^2^/L; with the increase in the treatment area, the conductivity and pH of the solution are gradually reduced, and the thickness and color are slow to change, which indicates that in the process of oxidizing and coloring the electrolytes in the service, the phenomenon of the “abnormal depletion” of copper ions has been improved. The phenomenon has been improved.

(3) After the introduction of negative pulse, in the process of black coating formation of magnesium-based micro-arc oxidation, the average consumption of copper is 45.3 mg/L·dm^2^, which is only 26% of the consumption under the unipolar condition; after the introduction of negative pulse, it can significantly improve the “abnormal consumption” of copper ions and increase the service life of the electrolytes. The change in the electric field by the negative pulse makes the Cu ions enriched at the cathode migrate to the anode and reduces the reduction and precipitation of Cu ions at the cathode.

## Figures and Tables

**Table 1:** Surface EDS composition analysis of micro-arc oxidized coatings (wt. %).

Concentration (g/L)	Percentage of Element Content (wt. %)				
	Mg	O	Si	Cu	F
D1	52.91	33.33	7.89	3.39	2.48
D2	55.36	35.08	7.29	0.01	2.27
D3	53.49	33.33	7.59	3.70	1.88
D4	56.14	35.48	6.31	0.44	1.62

**Table 2:** Content of elemental Cu before and after electrolyte failure in different power modes.

Power Mode	Pre-Invalidation (Cu mg/L)	Post-Invalidation (Cu mg/L)
unipolar	1495	448
bipolar	1495	770

## Data Availability

Data are contained within the article.
